# Population prevalence and inheritance pattern of recurrent CNVs associated with neurodevelopmental disorders in 12,252 newborns and their parents

**DOI:** 10.1038/s41431-020-00707-7

**Published:** 2020-08-10

**Authors:** Dinka Smajlagić, Ksenia Lavrichenko, Siren Berland, Øyvind Helgeland, Gun Peggy Knudsen, Marc Vaudel, Jan Haavik, Per Morten Knappskog, Pål Rasmus Njølstad, Gunnar Houge, Stefan Johansson

**Affiliations:** 1 Department of Clinical Science, University of Bergen, Bergen, Norway; 2 Department of Medical Genetics, Haukeland University Hospital, Bergen, Norway; 3 Computational Biology Unit, Department of Informatics, University of Bergen, Bergen, Norway; 4 Department of Genetics and Bioinformatics, Norwegian Institute of Public Health, Oslo, Norway; 5 Department of Biomedicine, University of Bergen, Bergen, Norway; 6 Division of Psychiatry, Haukeland University Hospital, Bergen, Norway; 7 Department of Pediatrics and Adolescents, Haukeland University Hospital, Bergen, 5021 Norway

**Keywords:** Neurodevelopmental disorders, Medical genetics, Genomics

## Abstract

Recurrent copy number variations (CNVs) are common causes of neurodevelopmental disorders (NDDs) and associated with a range of psychiatric traits. These CNVs occur at defined genomic regions that are particularly prone to recurrent deletions and duplications and often exhibit variable expressivity and incomplete penetrance. Robust estimates of the population prevalence and inheritance pattern of recurrent CNVs associated with neurodevelopmental disorders (NDD CNVs) are lacking. Here we perform array-based CNV calling in 12,252 mother–father–child trios from the Norwegian Mother, Father, and Child Cohort Study (MoBa) and analyse the inheritance pattern of 26 recurrent NDD CNVs in 13 genomic regions. We estimate the total prevalence of recurrent NDD CNVs (duplications and deletions) in live-born children to 0.48% (95% C.I.: 0.37–0.62%), i.e., ~1 in 200 newborns has either a deletion or duplication in these NDDs associated regions. Approximately a third of the newborn recurrent NDD CNVs (34%, *N* = 20/59) are de novo variants. We provide prevalence estimates and inheritance information for each of the 26 NDD CNVs and find higher prevalence than previously reported for 1q21.1 deletions (~1:2000), 15q11.2 duplications (~1:4000), 15q13.3 microdeletions (~1:2500), 16p11.2 proximal microdeletions (~1:2000) and 17q12 deletions (~1:4000) and lower than previously reported prevalence for the 22q11.2 deletion (~1:12,000). In conclusion, our analysis of an unselected and representative population of newborns and their parents provides a clearer picture of the rate of recurrent microdeletions/duplications implicated in neurodevelopmental delay. These results will provide an important resource for genetic diagnostics and counseling.

## Background

Copy number variants (CNVs) comprise a substantial fraction of human genetic variation and their role in disease has been studied extensively [1–3]. Recurrent CNVs have been implicated in a range of rare genomic disorders and neurodevelopmental traits, e.g., 1q21.1 microdeletion- and microduplication syndrome, Williams-Beuren syndrome, Prader–Willi/Angelman syndrome, 16p11.2 microdeletion- and microduplication syndrome, and DiGeorge syndrome [4].

Specific regions of the genome are particularly prone to recurrent deletions and duplications, typically through a process characterized by nonallelic homologous recombination (NAHR) between region specific low-copy repeats (LCRs) [5]. Many such recurrent CNVs have been found to cause genomic disorders, each characterized by distinct clinical features but with variable expressivity and incomplete penetrance [6]. Throughout the paper, we will hereafter refer to this set of CNVs as recurrent CNVs associated with neurodevelopmental disorders (NDD CNVs).

Albeit genomic disorders are clearly enriched in clinically ascertained samples and firmly established to increase the risk of neurodevelopmental disorders (NDDs), the true prevalence and thus penetrance in the general population are still uncertain for many CNVs [7–12]. Only a few population-based studies have investigated this to date, and there is a great need for unbiased prevalence estimates [13–15]. To our knowledge, the largest population study on recurrent CNVs was performed in the UK Biobank, a population cohort with participants aged 40–69 years with a participation rate of only 5% [16]. Due to ascertainment bias in most of these studies, it is not known to what degree these prevalence estimates represent the whole population, and the absence of parental CNV data makes it impossible to infer their inheritance pattern. The Norwegian Mother, Father, and Child Cohort Study (MoBa) is a population-based pregnancy cohort study that overcomes some of these challenges. MoBa was conducted by the Norwegian Institute of Public Health that recruited pregnant mothers from all over Norway from 1999 to 2008. DNA was obtained from both parents and children and the cohort now includes data from more than 114,000 births [17]. Thus, high participation rate, relatively low ascertainment bias, and the child–parent trio (i.e., mother–father–child) design together with large sample size make MoBa a unique resource for improving our understanding of the population prevalence and inheritance pattern for these recurrent CNVs.

In this study, we performed CNV analysis in 12,252 MoBa trios and provide comprehensive analyses of the prevalence, inheritance pattern and de novo rate of recurrent copy number deletions and duplications implicated in neurodevelopmental genomic disorders, i.e., microdeletions and microduplications in the 1q21.1, 3q29, 7q11.23 (Williams-Beuren syndrome), 15q11.2 (Prader–Willi/Angelman syndrome (PW/AS)), 15q13.3, 16p11.2 distal and proximal, 17p13.3 (Miller-Dieker syndrome), 17p11.2 (Smith-Magenis and Potocki-Lupski syndrome), 17q12, 17q21.31 (Koolen-de Vries syndrome), and 22q11.2 distal and proximal regions.

## Materials and methods

### The Norwegian Mother, Father, and Child Cohort Study (MoBa)

The Norwegian Mother, Father, and Child Cohort Study (MoBa) is a population-based pregnancy cohort study conducted by the Norwegian Institute of Public Health [17]. Participants were recruited from all over Norway from 1999 to 2008. The pregnant women were invited to participate before the 17th week of pregnancy, and 41% of women consented to participation. The cohort now includes 114,500 children, 95,200 mothers and 75,200 fathers. The current study is based on version 9 of the quality-assured data files released for research. The establishment of MoBa and initial data collection were based on a license from the Norwegian Data Protection Agency and approval from The Regional Committees for Medical and Health Research Ethics. The MoBa cohort is based on regulations of the Norwegian Health Registry Act. The current study was approved by The Regional Committees for Medical and Health Research Ethics (2015/2055).

### Genotyping, quality control, CNV calling, and filtering

SNP-based genotyping and quality control (QC) have been described elsewhere [18]. In summary, MoBa1 data (9508 trios) were genotyped using Illumina’s HumanCoreExome-12 v.1.1 and HumanCoreExome-24 v.1.0 arrays, while MoBa2 data (5274 trios) were genotyped on Illumina’s Global Screening Array v.1.0. Variants with call rate <98% and out of Hardy–Weinberg equilibrium (*P* < 1.00E−06) were excluded. Individuals with call rate <98%, excessive heterozygosity (>4 standard deviations above the mean heterozygosity in the sample) and non-Norwegian ancestry were removed as well. Pairs of individuals with PI_HAT > 0.1 in identity by descent calculations were QC-ed by keeping a random individual and removing the other one, in each pair.

CNV-based genotyping and QC. The Log R Ratio (LRR) and B Allele Frequency (BAF) values were extracted using GenomeStudio (version v.2011.1 for discovery and v.2.0.3 for replication) (https://www.illumina.com/techniques/microarrays/array-data-analysis-experimental-design/genomestudio.html). The CNVs were called using PennCNV, followed by the PennCNV trio module (version 1.0.3 for discovery and 1.0.4 for replication) [19] using default settings. The merging of adjacent CNV fragments was done using clean_cnv.pl script of PennCNV suit, controlling for the efficiency of merging by examining bed tracks of CNV segments before and after the merging in the UCSC Genome Browser. Sample-level filtering was done with recommended parameters, e.g., the Log R Ratio standard deviation (LRR_SD) < 0.3, BAF_drift < 0.001, absolute value of wave factor (|WF|) < 0.05, number of CNV calls < 100 (MoBa1) and <130 (MoBa2) to account for slight differences in array densities. After the QC only high-quality trios in which all three individuals passed the above requirements were taken forward for further filtering (*N* = 7986 trios (Moba1), *N* = 4266 trios (MoBa2), median LRR_SD = 0.103, and median number of CNV calls = 10).

The frequency filtering was done using PLINK version 1.07 [20] removing all calls that had a frequency >1% in the parental set only (unrelated individuals). Next, all calls overlapping centromeric or telomeric regions as well as known copy number susceptible loci, e.g., immunoglobulin, were removed. Among the remaining CNVs, only calls spanning at least 100 kilo base pair (kb) and 10 markers were retained for downstream analyses.

### Definition and identification of recurrent NDD CNVs

After CNVs were called, merged and filtered based on frequency and size, only CNVs located within regions of known recurrent genomic syndromes were taken forward for analysis. The intersection was done using BEDTOOLS version 2.27.1 [21]. All candidate NDD CNVs were visualized and manually inspected using scatter plots of raw intensity value points (LRR and BAF values) along the genomic axis, for all individuals in each trio in putative CNVs and flanking regions, with help of the ggpubr package [22] to: (1) assess the evidence for a CNV, (2) identify the inheritance pattern, and (3) correct any erroneous breakpoints (one duplication of 22q11.2 in offspring number 53 (o53) was extended based on the signal in the flanks consistent with a continuation of a CNV). One offspring CNV (a duplication of 15q11.2–q13.1 in offspring number 13 (o13)) showed BAF and LRR distributions consistent with a mosaic copy number gain of maternal origin as one possibility.

The parental origin of de novo events was possible to resolve unambiguously for all but one event (a duplication of 16p11.2. distal in offspring number 33 (o33)). For deletions we used the infer_snp_allele.pl script from the PennCNV package [19] and for duplications we developed an inhouse script that assessed the parental origin of all three alleles at each marker as described elsewhere [23].

CNVs spanning at least 50% of the reference NDD region included in the statistical analyses in the study (Supplementary File 1 for coordinates according to the Genome Reference Consortium Human Build 37 (GRCh37) and Supplementary File 2 for visualization of all CNVs included in the final analyses). The final set of NDD CNVs was deposited to dbVar under accession nstd192.

### Prevalence estimates

Prevalence was calculated as detailed in Eq. (1):

1 $$\frac{{NCNVs}}{{Ntrios}},$$

where *NCNVs* refers to the total number of recurrent NDD CNVs and *Ntrios* refers to the total number of complete trios that passed the QC. The Wilson score interval test was used to provide the 95% confidence intervals around the prevalence estimates. For some of the investigated recurrent CNVs, we found zero events among our 12,252 children. Using the Wilson score interval test, we estimate the 95% confidence interval for the prevalence for these CNVs to be between 0 and 0.0003.

Two proportions *Z*-test was used to test for the differences between the total number of deletions and total number of duplications. The transmission disequilibrium test was performed according to [24] using the total number of NDD CNVs in a mother and total number of maternally inherited NDD CNVs in a child and the total number of NDD CNVs in a father and total number of paternally inherited NDD CNVs in a child.

## Results

In this study we investigated recurrent CNVs that span chromosomal regions known to be associated with NDDs i.e., 1q21.1, 3q29, 7q11.23, 15q11.2–13.1, 15q13.3, 16p11.2 distal and proximal, 17p13.3, 17p11.2, 17q12, 17q21.31 and 22q11.2 distal and proximal (Supplementary File 3).

**Fig. 1:**
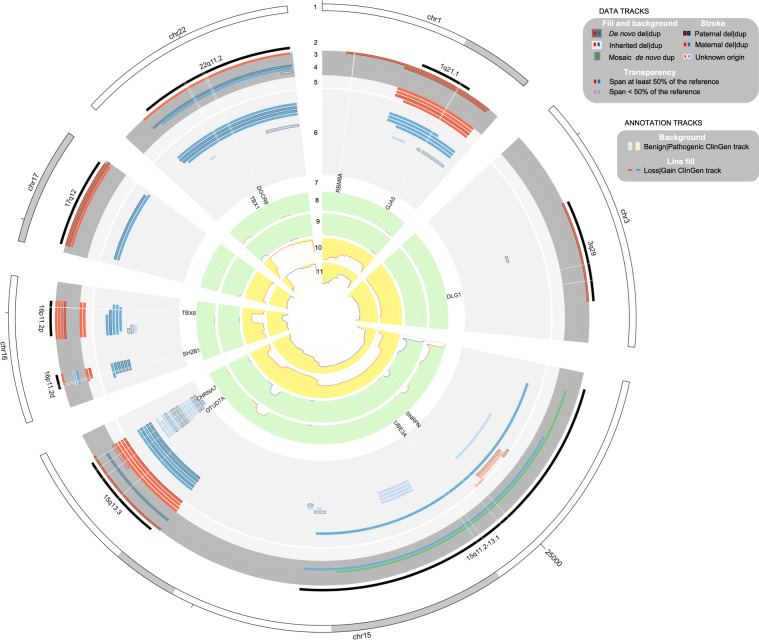
Circular plot of recurrent CNVs implicated in NDDs identified in MoBa trios. The tracks from outer to inner circles: ANN (annotation); DATA (our data): (1) ANN:Chromosomes  on an ideogram; (2) ANN:NDD-relevant reference intervals; (3) DATA:de novo deletions; (4) DATA:de novo duplications; (5) DATA:inherited deletions; (6) DATA:inherited duplications; (7) ANN:genes of interest; (8) ANN:ClinGen Benign Loss cumulative track; (9) ANN:ClinGen Benign Gain cumulative track; (10) ANN:ClinGen Pathogenic Loss cumulative track; (11) ANN:ClinGen Pathogenic Gain cumulative track.

The total number of Norwegian trios that passed QC was 12,252. After classification of CNVs and QC, 59 recurrent NDD CNVs were observed in the offspring (Supplementary File 2), of which 39 were identified as inherited and 20 were identified as de novo variants (Table 1, Fig. 1). This provided an estimate for the total prevalence of recurrent NDD CNVs in this birth cohort of 0.48% (95% confidence interval (C.I.): 0.37%, 0.62%) or in other words, 1 in 200 children was born with a recurrent deletion or duplication in these 13 regions known to be associated with NDDs.

**Table 1:** Cumulative prevalence estimates of recurrent NDD CNVs.

	Counts			Prev (95% C.I.)			Two proportions *Z*-test	
Recurrent CNVs	Del	Dup	Total	Del	Dup	Total	*Z* score	*P* value
All	25	34	59	20.4 (13.83, 30.11)	27.75 (19.87, 38.75)	48.16 (37.35, 62.06)	−1.17	0.24
Inherited	12	27	39	9.79 (5.6, 17.11)	22.04 (15.15, 32.04)	31.83 (23.3, 43.48)	−2.4	0.016
De novo	13	7	20	10.61 (6.2, 18.15)	5.71 (2.77, 11.79)	16.32 (10.57, 25.2)	1.34	0.18

*Del* copy number deletions, *Dup* copy number duplications, *Prev* prevalence in 10,000.

A total of 25 events were deletions while 34 were duplications. We observed a lower proportion of deletions compared to duplications (12 deletions vs. 27 duplications) among the inherited variants while the opposite trend was noticed among the de novo calls (13 de novo deletions vs. 7 de novo duplications). As can be seen in Table 1, only the difference observed between inherited deletions and duplication reached nominal significance (*P* = 0.02). Only one de novo CNV was consistent with a mosaic event, a 15q13.3 copy number gain of maternal origin (Supplementary File 2, offspring o13, page 4).

**Fig. 2:**
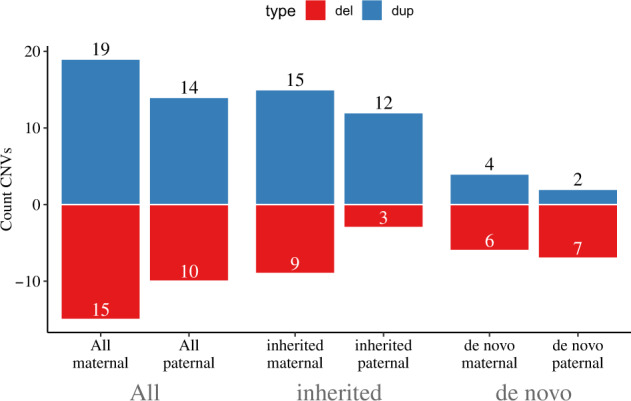
Parental origin of recurrent CNVs implicated in NDDs identified in MoBa trios. The total number of NDD CNVs with resolved parent-of-origin is 58 (for a single de novo duplication in 16p11.2 distal region it was not possible to resolve the inheritance status).

**Fig. 3:**
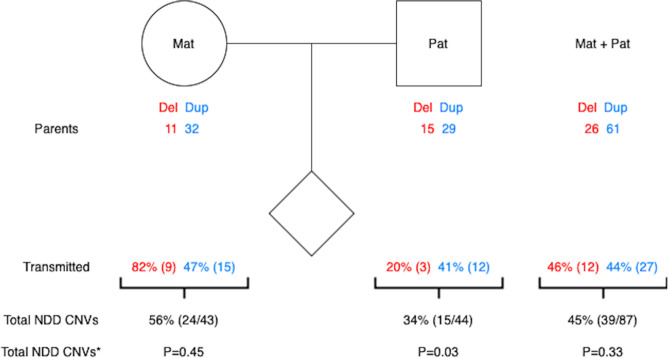
Transmission of recurrent CNVs implicated in NDDs identified in MoBa. Parents label indicates number of CNVs stratified on parental status, transmitted label indicates number of transmitted NDD CNVs. Mat maternal origin, Pat paternal origin, * indicates *P* values from transmission disequilibrium test with one degree of freedom.

Since MoBa is a study where both mother and father were genotyped together with the offspring, we were able to assess the parent-of-origin status of recurrent NDD CNVs (Fig. 2). We observed a higher rate of maternal variants among the inherited calls (24 vs. 15), contrary to de novo variants for which recurrent NDD CNVs occurred in equal numbers on both the maternally and paternally derived chromosomes (10 vs. 9) (Fig. 2). For one de novo duplication we were not able to assign the parent-of-origin status. We also assessed the number of recurrent NDD CNVs in mothers and fathers per region and calculated the cumulative transmission rate of maternal and paternal CNVs (55.81% vs. 34.09%) (Supplementary File 4 and Fig. 3). The overall prevalence (and their corresponding 95% C.I.) of events in mothers (0.35% (95% C.I.: 0.26%, 0.47%)) and fathers (0.36% (95% C.I.: 0.27%, 0.48%)) were similar, but lower than what was seen in children (0.48% (95% C.I.: 0.37%, 0.62%)).

### Prevalence estimates

We next calculated prevalence estimates (with their corresponding 95% C.I.) for each recurrent microdeletion and microduplication located in the 1q21.1, 3q29, 7q11.23, 15q11.2, 15q13.3, 16p11.2, 17p11.2, 17p13.3, 17q12, 17q21.31, and 22q11.2 regions (Table 2). We did not observe any events in the 7q11.23 (Williams-Beuren syndrome) region, 17p13.3 (Miller-Dieker syndrome) region, 17q21.31 (Koolen-de Vries syndrome) region, and 22q11.2 distal region, nor in the 17p11.2 region (Smith-Magenis and Potocki-Lupski syndromes) (Table 2).

**Table 2:** Prevalence estimates of recurrent NDD CNVs spanning 13 regions associated with neurodevelopmental disorders.

Recurrent CNVs			De novo CNVs			Maternal CNVs		Paternal CNVs		All CNVs	
Position (hg37)	Description	Length (Mb)	*N*	Prev (95% C.I.)	Inheritance status	*N*	Prev (95% C.I.)	*N*	Prev (95% C.I.)	*N*	Prev (95% C.I.)
1:146578858:147396590	1q21.1:Del	0.817732	2	1.63 (0.45, 5.95)	Paternal x2	3	2.45 (0.83, 7.2)	1	0.82 (0.14, 4.62)	6	4.9 (2.24, 10.68)
1:146578858:147396590	1q21.1:Dup	0.817732	0	0		4	3.26 (1.27, 8.39)	0	0	4	3.26 (1.27, 8.39)
3:195759926:197346552	3q29:Del	1.586626	1	0.82 (0.14, 4.62)	Paternal	0	0	0	0	1	0.82 (0.14, 4.62)
3:195759926:197346552	3q29:Dup	1.586626	0	0		0	0	0	0	0	0
7:72745828:74145433	7q11.23 (Williams-Beuren):Del	1.399605	0	0		0	0	0	0	0	0
7:72745828:74145433	7q11.23:Dup	1.399605	0	0		0	0	0	0	0	0
15:22754735:28570518	15q11.2–13.1 (Prader–Willi/Angelman):Del	5.815783	0	0		0	0	0	0	0	0
15:22754735:28570518	15q11.2–13.1:Dup*	5.815783	2	1.63 (0.45, 5.95)	Maternal x2*	1	0.82 (0.14, 4.62)	0	0	3	2.45 (0.83, 7.2)
15:31075791:32446853	15q13.3:Del	1.371062	1	0.82 (0.14, 4.62)	Paternal	3	2.45 (0.83, 7.2)	1	0.82 (0.14, 4.62)	5	4.08 (1.74, 9.55)
15:31075791:32446853	15q13.3:Dup	1.371062	1	0.82 (0.14, 4.62)	Paternal	2	1.63 (0.45, 5.95)	3	2.45 (0.83, 7.2)	6	4.9 (2.24, 10.68)
16:28823089:29046734	16p11.2 distal:Del	0.223645	1	0.82 (0.14, 4.62)	Maternal	1	0.82 (0.14, 4.62)	1	0.82 (0.14, 4.62)	3	2.45 (0.83, 7.2)
16:28823089:29046734	16p11.2 distal:Dup^§^	0.223645	2	1.63 (0.45, 5.95)	Maternal unknown^§^	1	0.82 (0.14, 4.62)	5	4.08 (1.74, 9.55)	8	6.53 (3.31, 12.88)
16:29650744:30195048	16p11.2 proximal:Del	0.544304	4	3.26 (1.27, 8.39)	Maternal x3 paternal	2	1.63 (0.45, 5.95)	0	0	6	4.9 (2.24, 10.68)
16:29650744:30195048	16p11.2 proximal:Dup	0.544304	0	0		4	3.26 (1.27, 8.39)	1	0.82 (0.14, 4.62)	5	4.08 (1.74, 9.55)
17:1247834:2588909	17p13.3 (YWHAE, PAFAH1B, Miller-Dieker syndrome):Del	1.341075	0	0		0	0	0	0	0	0
17:1247834:2588909	17p13.3 (YWHAE, PAFAH1B1):Dup	1.341075	0	0		0	0	0	0	0	0
17:16811743:20216832	17p11.2 (Smith-Magenis):Del	3.405089	0	0		0	0	0	0	0	0
17:16811743:20216832	17p11.2 (Potocki-Lupski):Dup	3.405089	0	0		0	0	0	0	0	0
17:34816572:36215672	17q12:Del	1.3991	3	2.45 (0.83, 7.2)	Maternal paternal x2	0	0	0	0	3	2.45 (0.83, 7.2)
17:34816572:36215672	17q12:Dup	1.3991	0	0		1	0.82 (0.14, 4.62)	1	0.82 (0.14, 4.62)	2	1.63 (0.45, 5.95)
17:43706119:44165156	17q21.31 (Koolen-de Vries):Del	0.459037	0	0		0	0	0	0	0	0
17:43706119:44165156	17q21.31:Dup	0.459037	0	0		0	0	0	0	0	0
22:19024860:21469135	22q11.2 (DiGeorge):Del	2.444275	1	0.82 (0.14, 4.62)	Maternal	0	0	0	0	1	0.82 (0.14, 4.62)
22:19024860:21469135	22q11.2:Dup	2.444275	2	1.63 (0.45, 5.95)	Maternal paternal	2	1.63 (0.45, 5.95)	2	1.63 (0.45, 5.95)	6	4.9 (2.24, 10.68)
22:21920486:23652202	22q11.2 distal:Del	1.731716	0	0		0	0	0	0	0	0
22:21920486:23652202	22q11.2 distal:Dup	1.731716	0	0		0	0	0	0	0	0
	Total		20	16.32 (10.57, 25.2)		24	19.59 (13.17, 29.13)	15	12.24 (7.42, 20.19)	59	48.16 (37.35, 62.06)

*N* counts, *Prev* prevalence in 10,000.

*Indicates that one of the de novo duplications is mosaic and of maternal origin, ^§^indicates that we could not assess the origin of one de novo variant.

We noticed a tendency toward more maternally derived than paternally derived recurrent CNVs for the 1q21.1 and typical 0.6 mega base pair (Mb)/proximal 16p11.2 microdeletion and microduplication (Tables 3, 4, respectively). A total of seven maternal and three paternal calls were detected in the 1q21.1 region while nine maternal and two paternal calls were detected in the 16p11.2 region (Tables 3, 4). All of the seven maternally derived 1q21.1 CNVs and 2/3 of the paternally derived events were inherited, and for the 0.6 Mb/proximal 16p11.2 region, 6/9 of the maternally derived and 1/2 of the paternally derived CNVs were inherited.

**Table 3:** Prevalence estimates and inheritance status of recurrent NDD CNVs spanning the 1q21.1 region.

1q21.1								
	De novo		Inherited		Total			
	Mat	Pat	Mat	Pat	Mat	Prev (95% C.I.)	Pat	Prev (95% C.I.)
Del	0	2	3	1	3	2.45 (0.83, 7.2)	3	2.45 (0.83, 7.2)
Dup	0	0	4	0	4	3.26 (1.27, 8.39)	0	0
TOTAL	0	2	7	1	7	5.71 (2.77, 11.79)	3	2.45 (0.83, 7.2)

*Mat* maternally derived, *Pat* paternally derived, *Prev* prevalence in 10,000, *Del* copy number deletions, *Dup* copy number duplications.

**Table 4:** Prevalence estimates and inheritance status of recurrent NDD CNVs spanning the 16p11.2 region.

16p11.2 proximal								
	De novo		Inherited		Total			
	Mat	Pat	Mat	Pat	Mat	Prev (95% C.I.)	Pat	Prev (95% C.I.)
Dels	3	1	2	0	5	4.08 (1.74, 9.55)	1	0.82 (0.14, 4.62)
Dups	0	0	4	1	4	3.26 (1.27, 8.39)	1	0.82 (0.14, 4.62)
TOTAL	3	1	6	1	9	7.35 (3.87, 13.96)	2	1.63 (0.45, 5.95)

*Mat* maternally derived, *Pat* paternally derived, *Prev* prevalence in 10000, *Del* copy number deletions, *Dup* copy number duplications.

## Discussion

In this study we estimated the prevalence and inheritance pattern of the recurrent CNVs implicated in neurodevelopmental disorders (NDD CNVs) among 12,252 mother–father–offspring trios from the Norwegian Mother, Father, and Child Cohort Study (MoBa). This is to our knowledge the first study using population-scale unselected parent–child trio CNV data. Here we estimated the total prevalence of recurrent NDD CNV duplications and deletions in Norwegian live-born children to 0.48% (95% C.I.: 0.37–0.62%). Approximately a third of the newborn recurrent NDD CNVs (34.0%, (20/59)) were de novo variants. Thus, our data suggest that ~1 in 600 children are born with a de novo event in these regions, setting the cumulative rate of recurrent NDD de novo CNVs to 0.16% per live-born child.

Having established the cumulative rate of NDD CNVs in children we were also able to compare these numbers with their parents. Interestingly, mothers and fathers have similar overall frequencies of recurrent NDD CNVs (43 vs. 44 for mothers and fathers respectively, *N* = 12,252 trios, 0.35% prevalence in mothers and 0.36% in fathers) and there is little, if no, overall transmission bias of these parental CNVs to their live-born children (45% transmission versus the expected 50% under the assumption of no selection operating on these CNVs (*P* = 0.33)). However, from fathers only, this number drops to 34% (15 of 44, *P* = 0.03). Hence among inherited events, 61.5% are inherited from mothers (25 of 39) (Table 3). This suggests that negative selection might be acting more strongly on the fecundity of paternal NDD CNV carriers, albeit considerably larger numbers are needed before firm conclusions can be drawn.

Among newborns, there was a significantly lower rate of inherited deletions compared to inherited duplications (12 vs. 27, *P* = 0.02, Table 1). This is consistent with an overall generally milder expressivity (i.e., lower severity) of duplications [4, 25, 26]. The same trend was not seen for de novo events for which there were slightly more deletions than duplications (13 vs. 6, *P* = 0.11). Thus, both the total frequency of events (59 in newborns vs. 43 in mothers and 44 in fathers) and the ratio of deletions vs. duplications were higher in newborns than in their parents. The 35% higher frequency of NDD CNVs in newborns compared to parents entering into the study illustrates the selection that is acting against the group of recurrent NDD CNVs investigated in this study.

Several studies have shown that de novo CNVs are more often of paternal origin [27]. We do not observe this tendency for this particular group of recurrent NDD CNVs (10 maternal vs. 9 paternal). Albeit the numbers are small, our data supports a few previous studies suggesting that for some recurrent NAHR-mediated CNVs, local gender specific recombination rates may be the determining factor for putative gender bias [23, 28].

Below we summarize and discuss the key observations for each individual region tested.

*1q21.1 microdeletion/microduplications* are known to be associated with learning problems and sometimes intellectual disability (ID), autism spectrum disorders (ASD), schizophrenia (SCZ), and attention deficit hyperactivity disorder (ADHD). Previous estimations have indicated that the prevalence for deletions is between 1 in 4000 and 1 in 6800 among healthy adult controls [4, 16, 29]. Our results suggest that in newborns, the number of deletion carriers is ~1 in 2000 (i.e., 0.05%) (Table 2), which is more common than what has been previously thought. Our data for the corresponding reciprocal duplication (prevalence of ~3 in 10,000) is however in line with previous estimates in the same adult control samples. This supports the statement that individuals with the 1q21.1 microduplication have less symptoms and are more likely to participate in biobank studies. Moreover, two out of six deletions are de novo. Of the inherited variants, it is notable that only one out of nine paternal duplications and deletions was transmitted to the offspring (Supplementary File 4). We are however not aware of previous claims of reduced paternal transmission for 1q21.1 microdeletion/duplications and this is likely a chance finding.

*15q.11.2–13.1 microdeletions* can cause Prader–Willi or Angelman syndrome, two neurobehavioral disorders caused by deletions of reciprocal imprinted genes. We did not observe any deletions in this region. However, we detected three maternally derived *15q.11.2–13.1 duplications* (one constitutional de novo duplication, one mosaic de novo gain in the offspring, and one duplication transmitted from the mother). The 15q duplication syndrome and related disorders are caused by maternally derived copy number gains at the PW/AS critical region and are associated with hypotonia, ASD, language delay and mild to moderate ID, even if the carrier mother is phenotypically normal [30]. Extra copies of this locus can result both from direct duplications due to unequal crossovers between the flanking LCRs, or LCR-based generation of an isodicentric supernumerary chromosome 15 (idic(15)) containing the PW/AS locus [31]. In the latter case, the locus copy number would normally be four. Two of the events identified here are clearly direct duplications. However, it is possible that the third—a mosaic gain (Supplementary File 2, offspring o13—page 4 top right), represents a mosaic form of idic(15).

*15q13.3 microdeletions* and the reciprocal *duplication* are also associated with learning problems and sometimes ID [32]. Some individuals with these CNVs experience developmental problems in social interaction and communication such as speech delay (as seen in patients with ASD) and a wide range of behavioral problems such as aggression, impulsive behavior and hyperactivity [33]. In the 15q13.3 region we found considerably higher prevalence for deletions than what has been estimated previously. Our data indicates that about 1 in 2500 children is born with the deletion compared to previous estimates at around 1 in 40,000 [32]. We also note that among our MoBa parents, the prevalence is similarly high (9 deletions in 24,502 parents: 0.04% prevalence, Supplementary File 4). The numbers are similar also for duplications. Thus, these data suggest that 15q13.3 microdeletions/duplications are associated with lower penetrance and/or milder clinical presentation than previously appreciated. Although the primary scope of this study is on the classical full-length recurrent NDD CNVs, we presented data on the much smaller “nested” CNV mainly restricted to the *CHRNA7* and *OTUD7A* genes in a separate track in Fig. 1 [34, 35]. This CNV is often seen in clinical diagnostic testing, and our results confirm that this event is indeed more common, but restricted to duplications (14 duplications, all inherited) with only 1 deletion (de novo) being detected in our sample. This observation supports that this smaller duplication is phenotypically neutral.

*16p11.2 proximal (classical) microdeletions and duplications* (of the typical ~0.6 Mb size) are associated with a range of NDDs with very variable penetrance and presentation between individual carriers. Key characteristics are developmental delay (often mild ID in the case of deletions), especially in speech and language, and ASD, but carriers also have significantly increased risk of SCZ, ADHD, obesity (if deletions), and seizures [11, 36–38]. Previous reports have provided various estimates of the prevalence of the deletions. In the Icelandic population they found 3.5 deletions per 10,000 individuals [39]. We find a similar but slightly higher prevalence (5 in 10,000) with 4 out of 6 deletions being de novo. In contrast, all duplications in our study were inherited. Furthermore, 3 out of 4 de novo and 6 out of 7 inherited variants were of maternal origin (Table 4), indicating a maternal bias. This result provides further support to a previous study that showed that 89% of all 16p11.2 proximal de novo deletions and duplications in their clinical cohort were of maternal origin [23]. In contrast to their ascertained case cohort, we found a tendency of maternal bias for both de novo and inherited 16p11.2 deletions/duplications in newborns (Table 4). This suggests that there might be a negative selection acting on paternal fecundity in addition to increased maternal de novo rate. This is consistent with results from Iceland showing that subjects carrying the 16p11.2 deletion had significantly fewer children than the general population and this effect was most attenuated among males [39].

*16p11.2 distal microdeletions and duplications* have been associated with learning problems, ASD, and obesity with variable phenotype and most likely relatively low penetrance [40, 41]. We observed 3 deletions spanning this region, of which 1 was de novo. The prevalence (~1:4000) was higher than what has been observed in other studies, for example in the UK biobank (~1:10,000) [16]. For the reciprocal 16p11.2 distal microduplications, we detected 8 duplications (2 de novo) in this region, which is again higher than suggested previously, indicating that approximately 1 in 1500 people carries this microduplication.

Both *17q12 microdeletions and duplications* can cause syndromes with variable symptoms even among affected members of the same family. The common abnormalities associated with the 17q12 deletions are problems with kidneys and urinary system, including kidney cysts and maturity-onset diabetes of the young type 5 (MODY5), hence 17q12 syndrome is also known as renal cysts and diabetes syndrome [42]. 17q12 microdeletions are also associated with developmental delay and psychiatric disease of very variable penetrance [43]. Individuals with the 17q12 duplication syndrome might not exhibit any symptoms, or they might have delayed development and mild ID. For deletions, we found three in our sample, all de novos. This sets the prevalence estimate to 2.5 events per 10,000 newborns, again considerably higher than previous indirect estimates of the population prevalence (1/14,500 according to [29]) and in biobank studies (1 in 156,000 UK biobank participants) [16]. This may suggest that a considerable number of 17q12 deletion carriers remain undiagnosed. Duplications seem to be more commonly inherited [44] and our prevalence estimate of 2 in 10,000 is more in line with biobank data and is consistent with a milder phenotype.

*22q11.2 microdeletions* can cause a range of symptoms with variable expressivity previously described as DiGeorge syndrome and velocardiofacial syndrome (MIM:188400; MIM:192430). This microdeletion is reported as one of the most common recurrent deletions in clinical cohorts and has also been associated with a range of psychiatric disorders including SCZ and ADHD [45]. We found only 1 carrier (de novo) among the 12,252 offspring in this study. This is considerably lower than what is the commonly cited figures for the prevalence of the 22q11.2 deletion syndrome in the literature of more than 1 per 2000 newborns [45] to 1 in 4000 [46, 47]. Our results suggest that the true prevalence of the 22q11 deletion is lower, and more likely is around 1 in 12,000. The reciprocal duplication is more common in our study. We observed two de novo and four inherited events in 12,252 offspring. This is in line with a milder phenotype/lower penetrance for duplications at this locus.

The study has both strengths and limitations. Although we argue that MoBa as a birth cohort provides a good representation of the general population and thus allows for robust estimations of the CNV burden among newborns, there are still some limitations to this study. Invitations to participate were sent to 277,702 pregnant women before their appointment around 17th week of pregnancy [17] and 41% participated in the study. Thus, there is a possible selection bias related to recruitment. Potential self-selection bias in MoBa has been studied by comparing the prevalence estimates and associations of different exposure and outcome variables between mothers who decided to participate in MoBa and all women whose deliveries were registered in the Medical Birth Registry of Norway in 2000–2006 [48]. Young women (<25 years of age), women living alone and women with more than two children are less represented in MoBa than in the general population (relative deviation 30–45). Similar trends were observed for smokers and women with stillbirths and neonatal deaths (relative deviation 22–43%) [48]. These deviations may indicate possible socioeconomic gradient influencing prevalence estimates. This trend may be further exacerbated through our trio study design that requires active participation of both the father (blood for DNA extraction) and mother. Hence it is likely that there is a bias against putatively deleterious and impairing CNVs in the parental generation. Consequently, our estimates of the inherited events may be biased downwards compared to the total newborn population. However, while the participation rate (41%) may indicate some recruitment bias, it is still considerably higher than most other comparable studies, such as a rate of 5% in the UK Biobank study [16]. Furthermore, since mothers were recruited already at pregnancy week 17, the prevalence estimates of de novo events should be more robust against selection biases. And finally, unique to this study, MoBa recruited both mothers and fathers. This allowed us to determine the inheritance pattern and assess the parental origin of the recurrent NDD CNVs in children and to calculate the transmission rate of the events from mothers and fathers.

By taking advantage of the trio study design of MoBa, we have provided improved prevalence estimates and inheritance details of 26 recurrent microdeletions and duplications associated with NDDs and psychiatric traits. These results should provide an important resource for clinical genetic diagnostics and increased insight of the genomic properties of this important class of variants.

## Supplementary information

Supplementary file 1. Genomic coordinates for NDD CNVs identified in 12252 newborns from the MoBa cohort

Supplementary file 2. Signal intensity (LRR and BAF) scatter plots for NDD CNVs identified in 12,252 newborns from the MoBa cohort

Supplementary file 3. Counts of rare recurrent NDD CNVs in two MoBa sample batches.

Supplementary file 4. Prevalence estimates and transmission rates of rare recurrent NDD CNVs.

## Data Availability

Access to genotypes and phenotypes can be obtained by direct request to the Norwegian Institute of Public Health (https://www.fhi.no/en/studies/moba/for-forskere-artikler/gwas-data-from-moba/). The final set of NDD CNVs was deposited to dbVar under accession nstd192.
