# Supplementary material for: Population prevalence and inheritance pattern of recurrent CNVs associated with neurodevelopmental disorders in 12,252 newborns and their parents
Source: Eur J Hum Genet. 2020 Aug 10;29(1):205–15. doi: 10.1038/s41431-020-00707-7 (PMC7852900; doi:10.1038/s41431-020-00707-7)
Supplement: Supplementary file 2 — Signal intensity (LRR and BAF) scatter plots for NDD CNVs identified in 12,252 newborns from the MoBa cohort [file 41431_2020_707_MOESM2_ESM.pdf]

Locus 1q21.1 : del

Locus spans 818 Kbp and 229 probes

mb1:o1:2442Kbp(301 pb) mb1:o2:1495Kbp(253 pb) mb1:o3:1495Kbp(256 pb) mb1:o4:1495Kbp(256 pb) mb1:o5:1324Kbp(252 pb) mb2:o2:1477Kbp(256 pb)

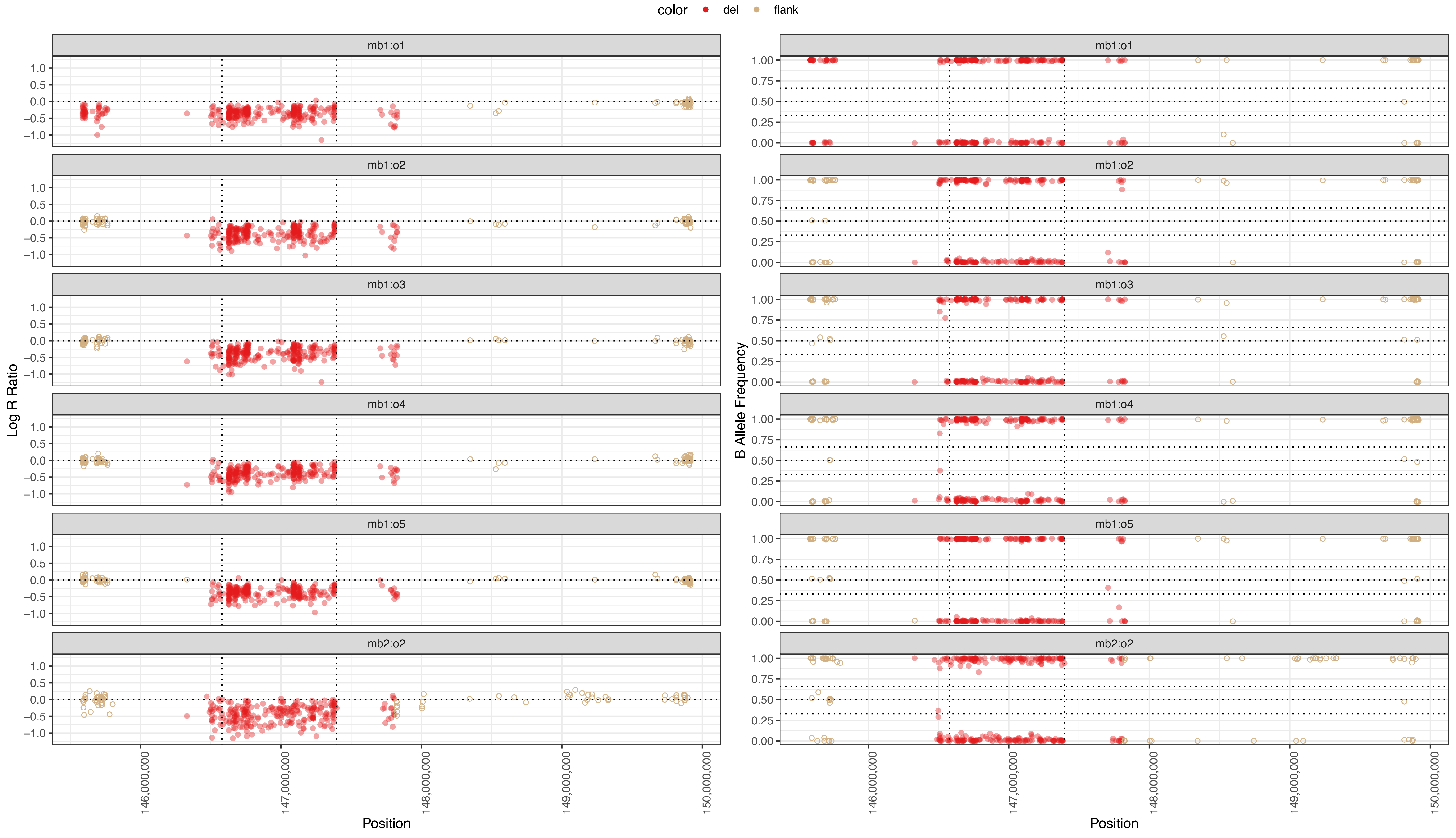

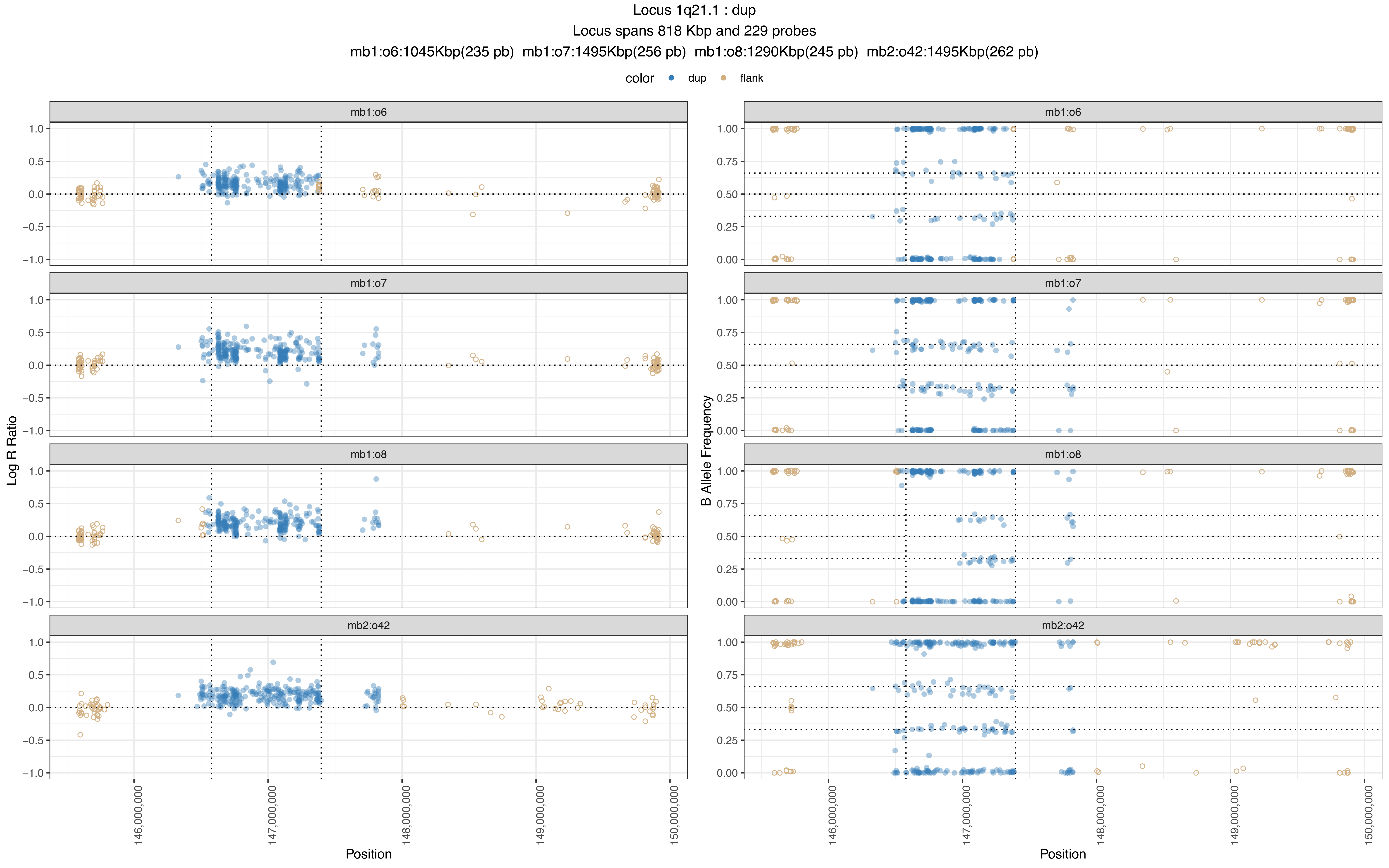
Locus 1q21.1 : dup
Locus spans 818 Kbp and 229 probes
mb1:o6:1045Kbp(235 pb) mb1:o7:1495Kbp(256 pb) mb1:o8:1290Kbp(245 pb) mb2:o42:1495Kbp(262 pb)
color dup flank
mb1:o6
mb1:o7
mb1:o8
mb2:o42
Log R Ratio
B Allele Frequency
Position
146,000,000
147,000,000
148,000,000
149,000,000
150,000,000

Locus 3q29 : del
Locus spans 1587 Kbp and 403 probes
mb1:o58:1596Kbp(406 pb)

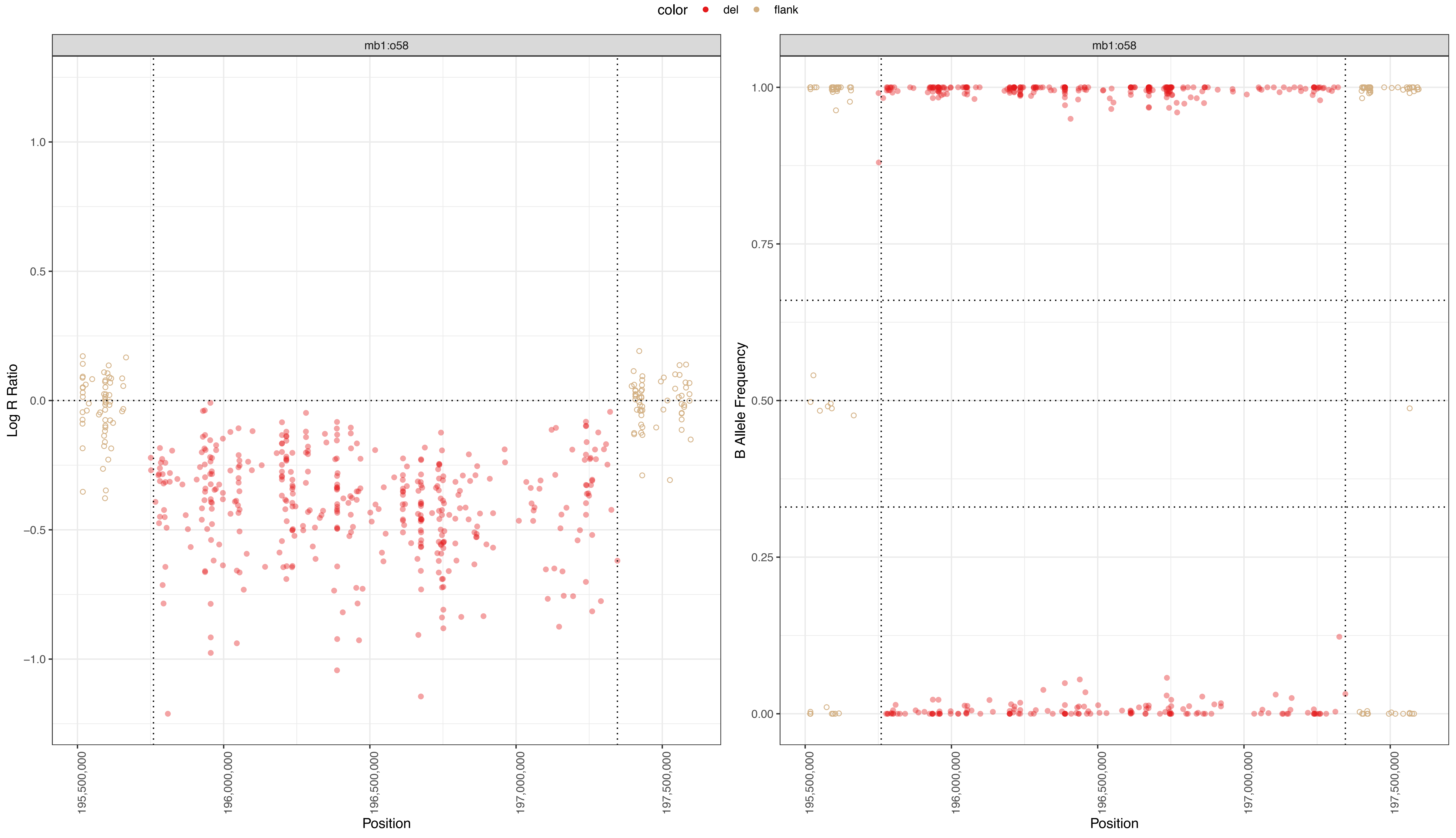

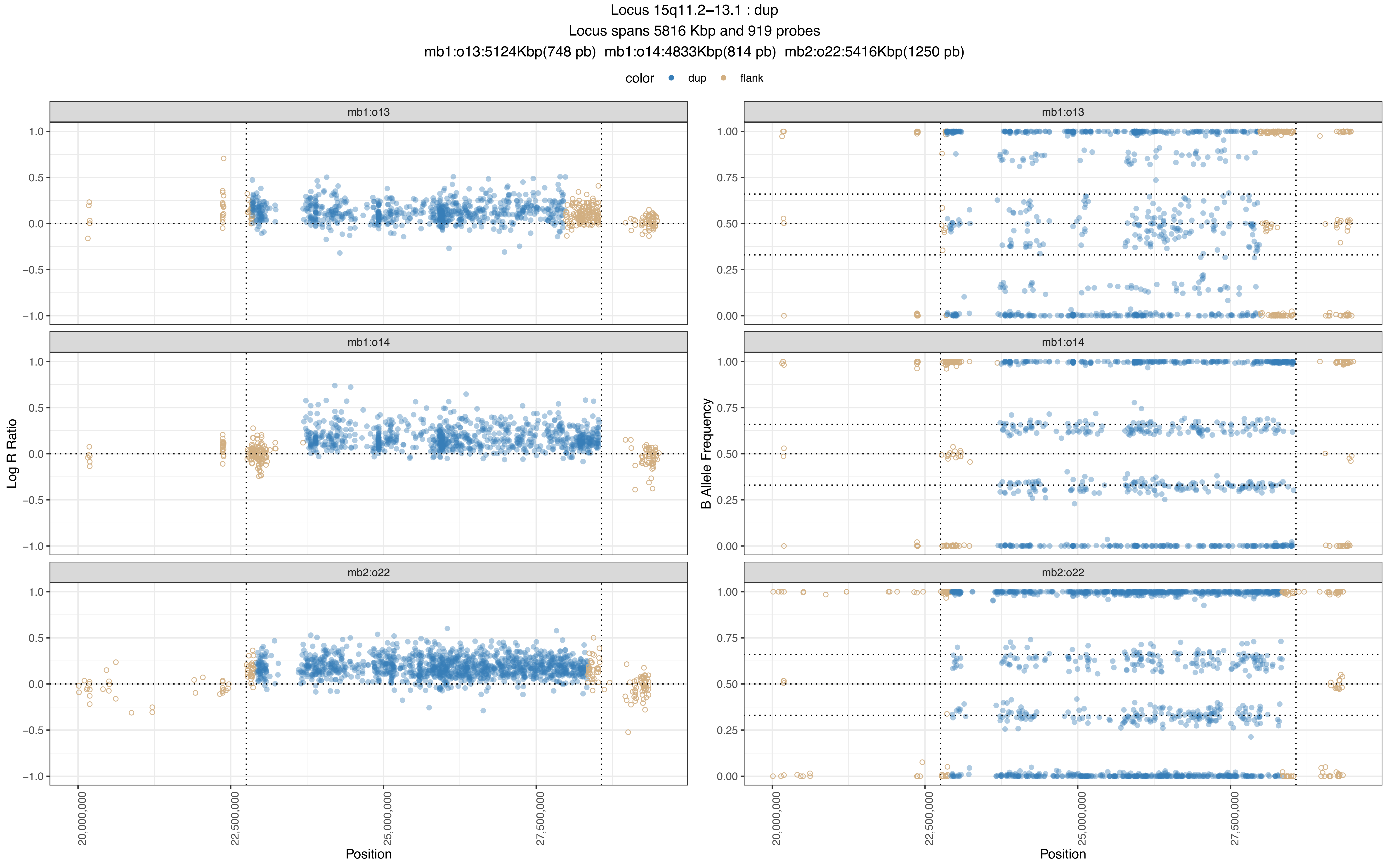

Locus 15q11.2−13.1 : dup
Locus spans 5816 Kbp and 919 probes
mb1:o13:5124Kbp(748 pb) mb1:o14:4833Kbp(814 pb) mb2:o22:5416Kbp(1250 pb)
color dup flank
mb1:o13
mb1:o14
mb2:o22
Log R Ratio
B Allele Frequency
Position
20,000,000
22,500,000
25,000,000
27,500,000

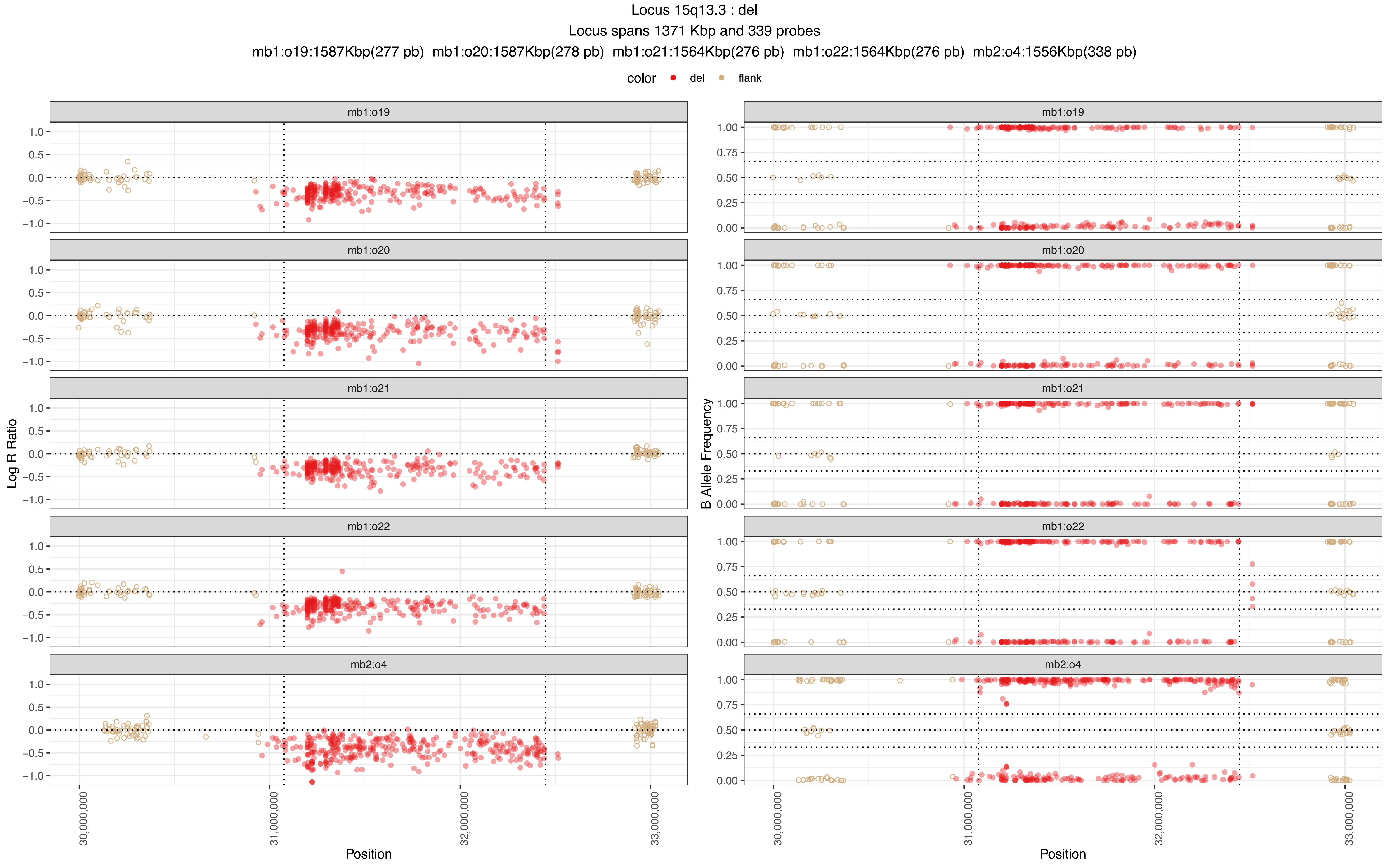
Locus 15q13.3 : del
Locus spans 1371 Kbp and 339 probes
mb1:o19:1587Kbp(277 pb) mb1:o20:1587Kbp(278 pb) mb1:o21:1564Kbp(276 pb) mb1:o22:1564Kbp(276 pb) mb2:o4:1556Kbp(338 pb)
color
del
flank
mb1:o19
mb1:o20
mb1:o21
mb1:o22
mb2:o4
Log R Ratio
B Allele Frequency
Position
30,000,000
31,000,000
32,000,000
33,000,000

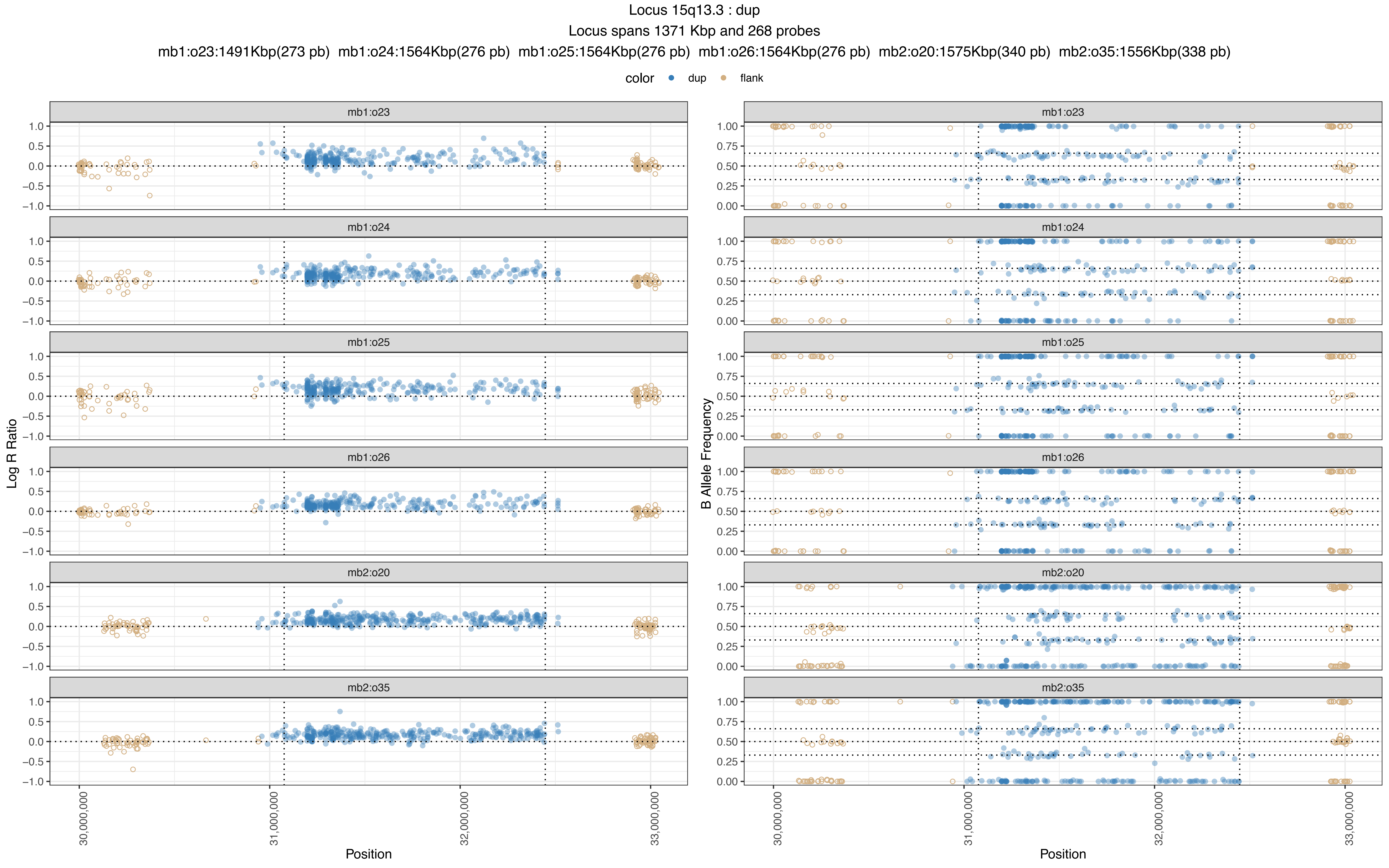

Locus 15q13.3 : dup
Locus spans 1371 Kbp and 268 probes
mb1:o23:1491Kbp(273 pb) mb1:o24:1564Kbp(276 pb) mb1:o25:1564Kbp(276 pb) mb1:o26:1564Kbp(276 pb) mb2:o20:1575Kbp(340 pb) mb2:o35:1556Kbp(338 pb)
color dup flank
mb1:o23
mb1:o24
mb1:o25
mb1:o26
mb2:o20
mb2:o35
Log R Ratio
B Allele Frequency
Position
30,000,000
31,000,000
32,000,000
33,000,000

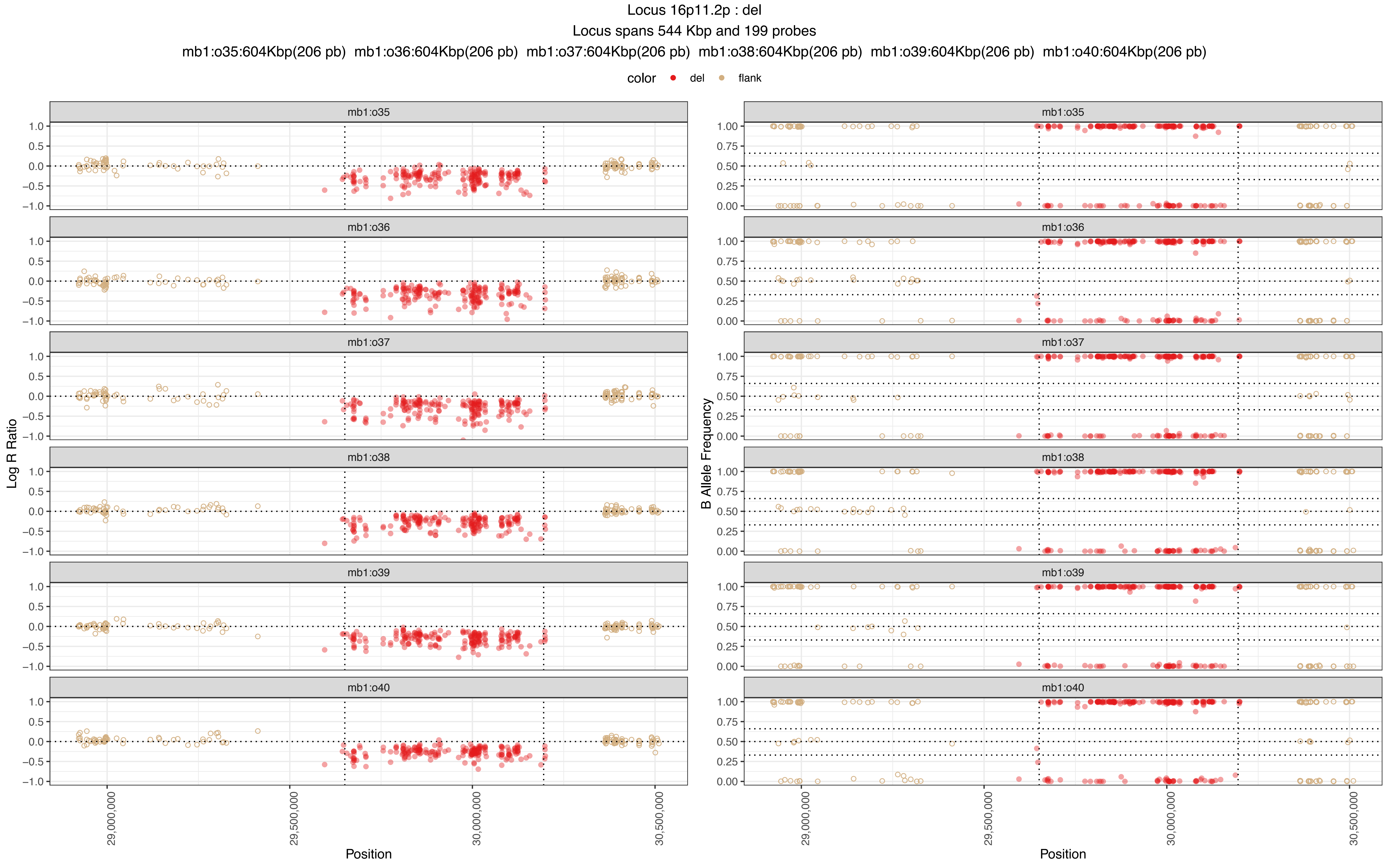
Locus 16p11.2p : del
Locus spans 544 Kbp and 199 probes
mb1:o35:604Kbp(206 pb) mb1:o36:604Kbp(206 pb) mb1:o37:604Kbp(206 pb) mb1:o38:604Kbp(206 pb) mb1:o39:604Kbp(206 pb) mb1:o40:604Kbp(206 pb)
color
del
flank
mb1:o35
mb1:o36
mb1:o37
mb1:o38
mb1:o39
mb1:o40
Log R Ratio
B Allele Frequency
Position
29,000,000
29,500,000
30,000,000
30,500,000

Locus 16p11.2p : dup

Locus spans 544 Kbp and 199 probes

mb1:o44:400Kbp(109 pb) mb1:o45:501Kbp(175 pb) mb1:o46:603Kbp(203 pb) mb1:o47:603Kbp(203 pb) mb2:o39:501Kbp(120 pb)

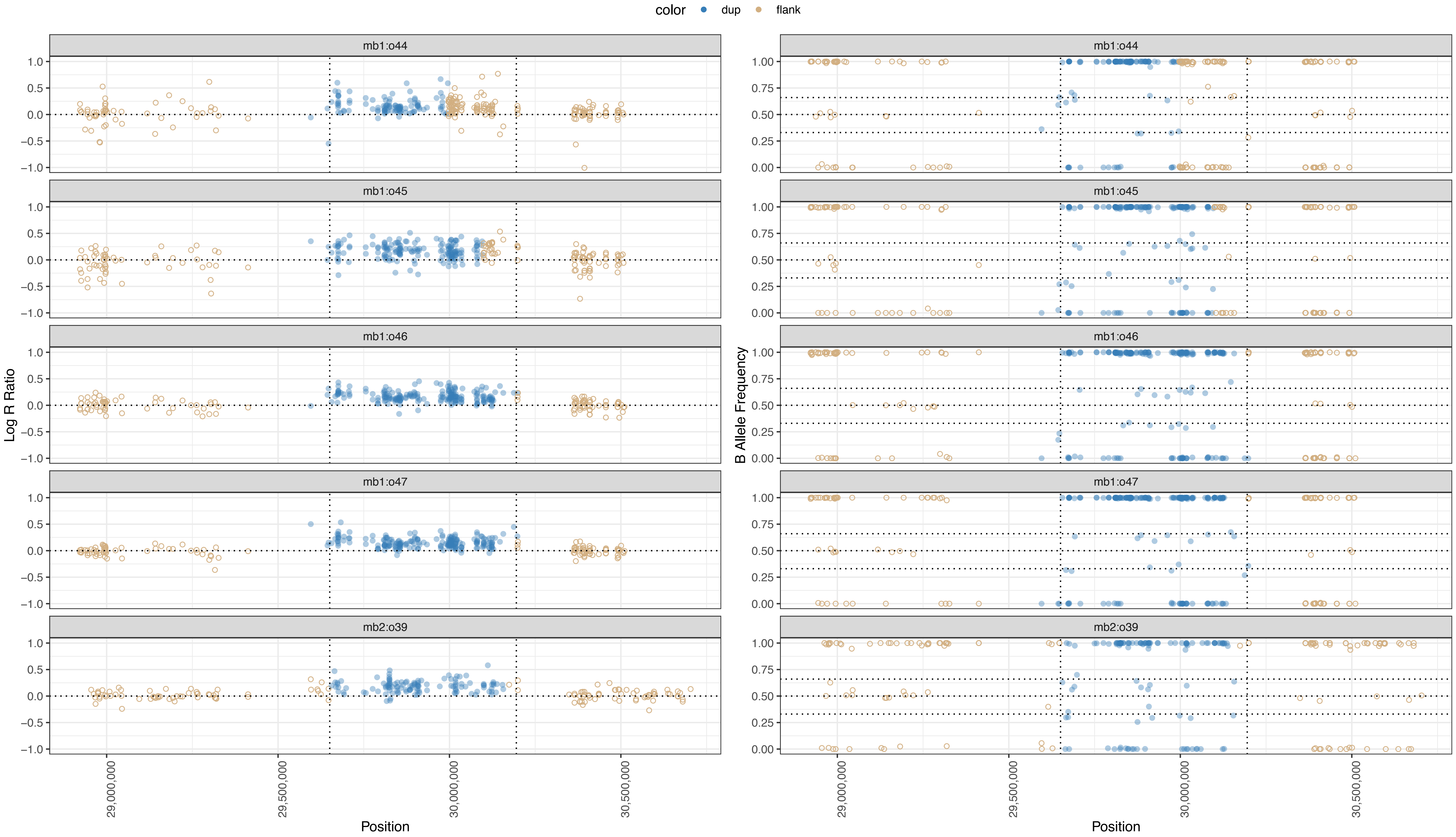

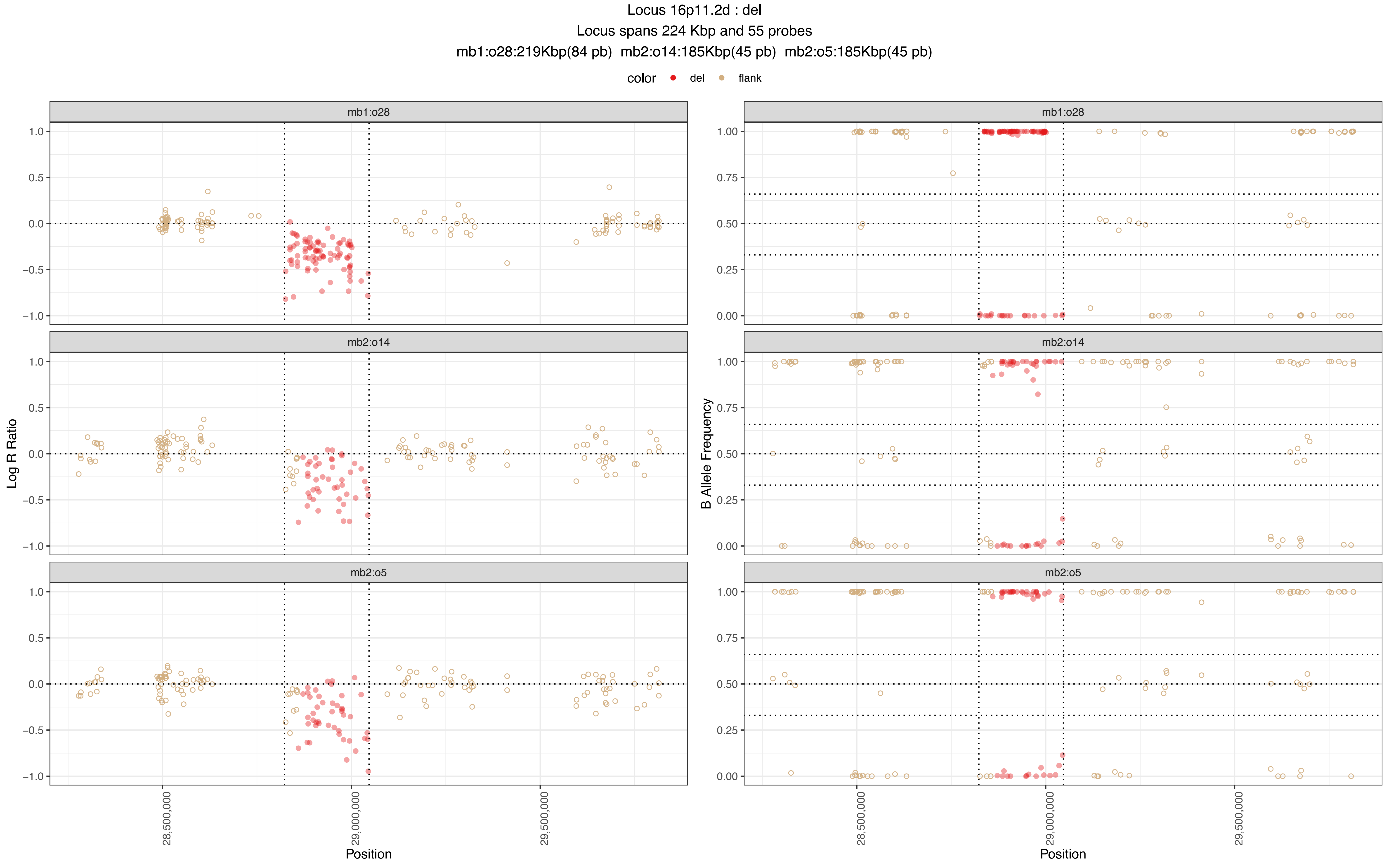

Locus 16p11.2d : del
Locus spans 224 Kbp and 55 probes
mb1:o28:219Kbp(84 pb) mb2:o14:185Kbp(45 pb) mb2:o5:185Kbp(45 pb)
color
del
flank
mb1:o28
mb2:o14
mb2:o5
Log R Ratio
B Allele Frequency
Position
28,500,000
29,000,000
29,500,000

Locus 16p11.2d : dup

Locus spans 224 Kbp and 84 probes

mb1:o29:290Kbp(85 pb) mb1:o30:219Kbp(84 pb) mb1:o31:219Kbp(84 pb) mb1:o32:219Kbp(84 pb) mb1:o33:219Kbp(84 pb) mb1:o34:219Kbp(84 pb) mb2:o21:219Kbp(55 pb) mb2:o6:219Kbp(55 pb)

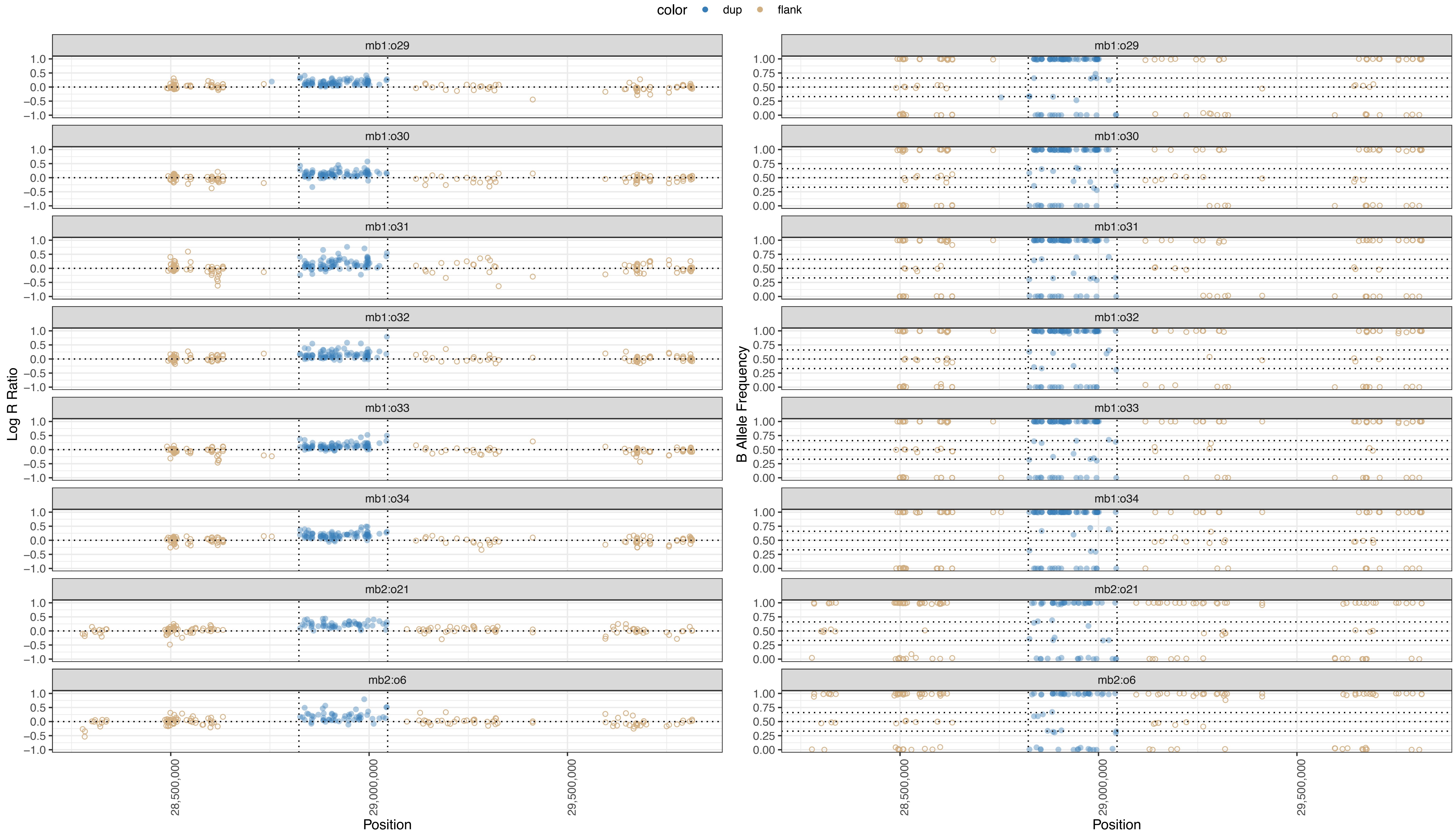

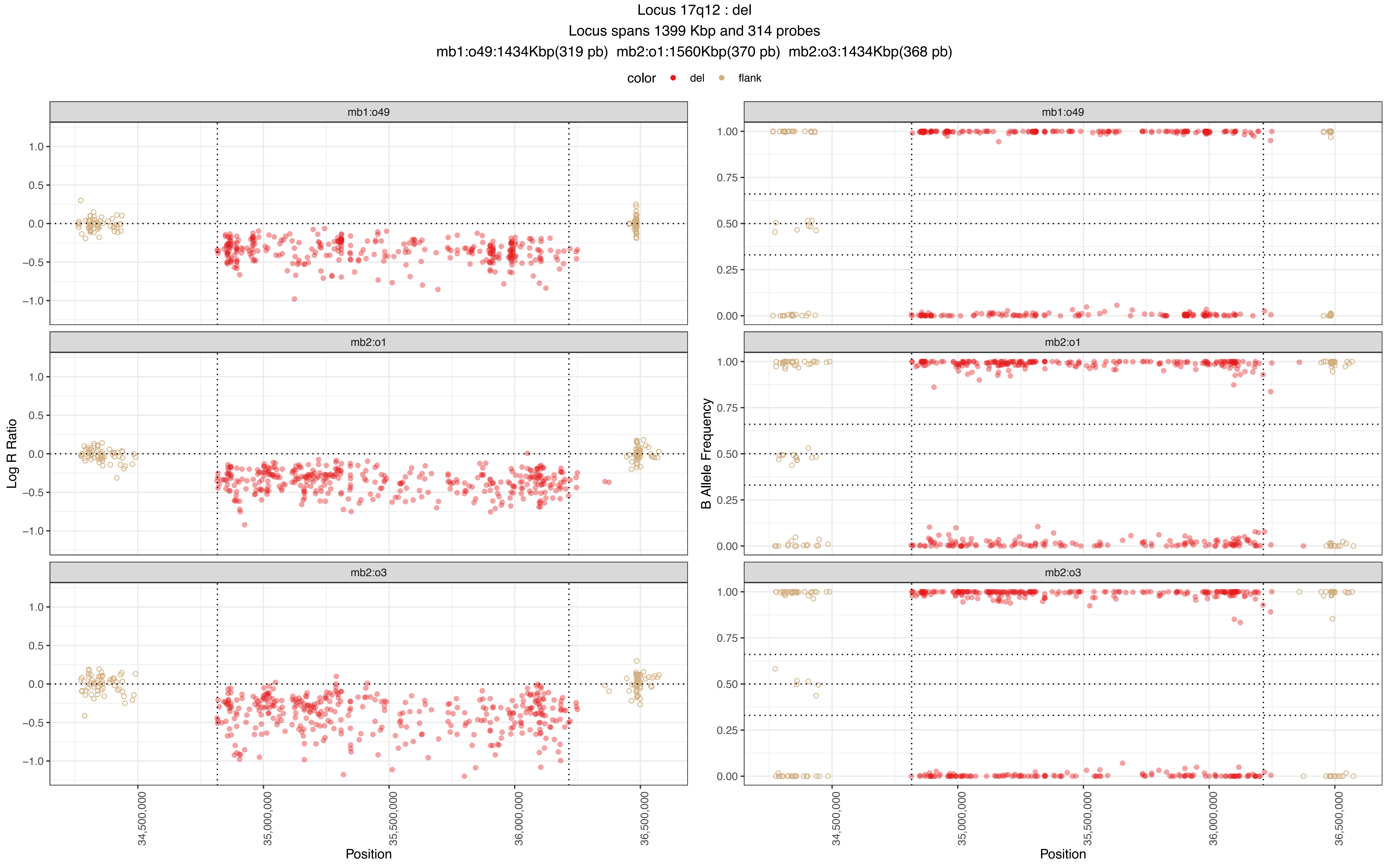
Locus 17q12 : del
Locus spans 1399 Kbp and 314 probes
mb1:o49:1434Kbp(319 pb) mb2:o1:1560Kbp(370 pb) mb2:o3:1434Kbp(368 pb)
color
del
flank
mb1:o49
mb2:o1
mb2:o3
Log R Ratio
B Allele Frequency
1.0
0.5
0.0
−0.5
−1.0
1.00
0.75
0.50
0.25
0.00
34,500,000
35,000,000
35,500,000
36,000,000
36,500,000
Position

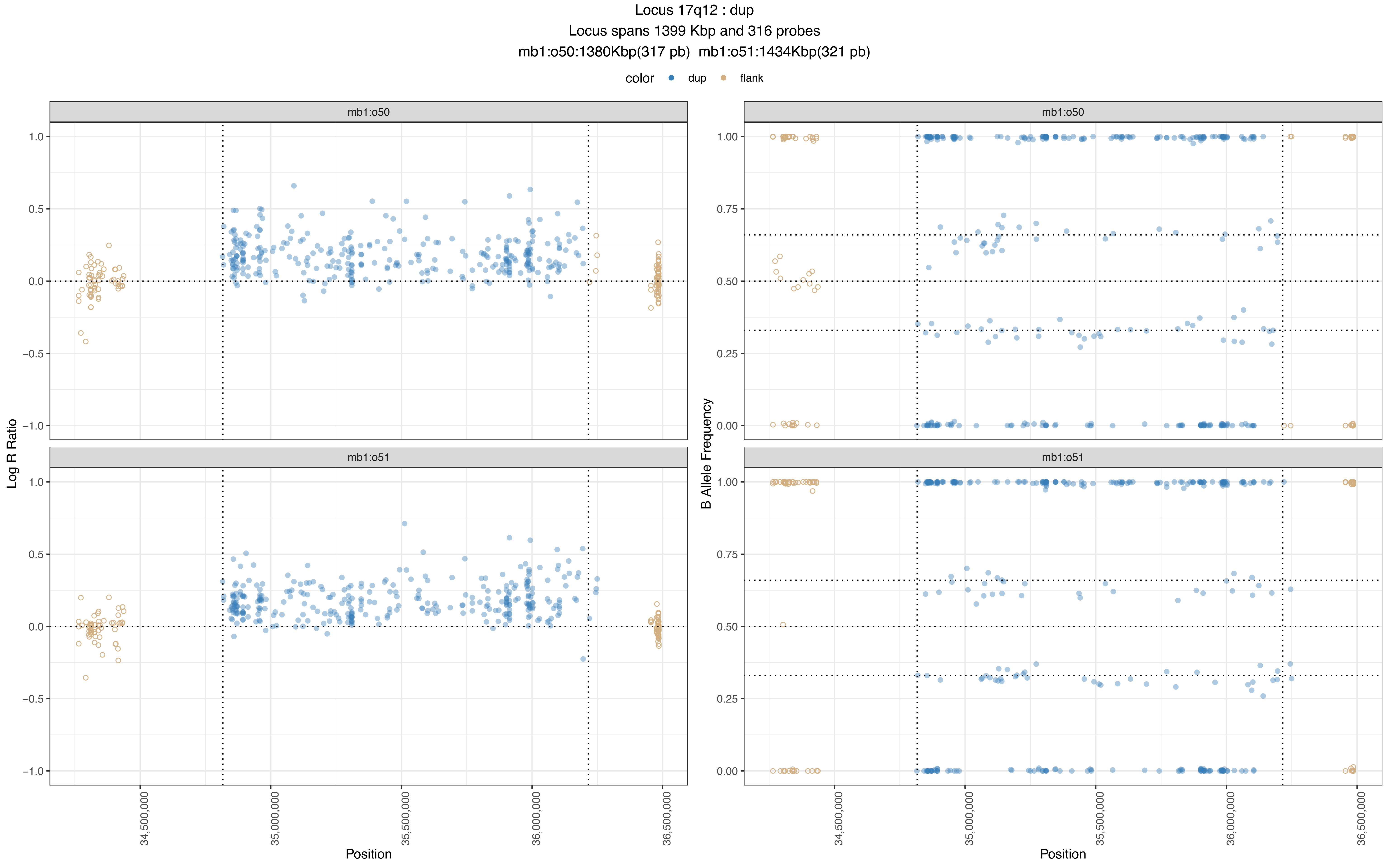

Locus 17q12 : dup
Locus spans 1399 Kbp and 316 probes
mb1:o50:1380Kbp(317 pb) mb1:o51:1434Kbp(321 pb)
color dup flank
mb1:o50
mb1:o51
Log R Ratio
B Allele Frequency
Position
34,500,000
35,000,000
35,500,000
36,000,000
36,500,000

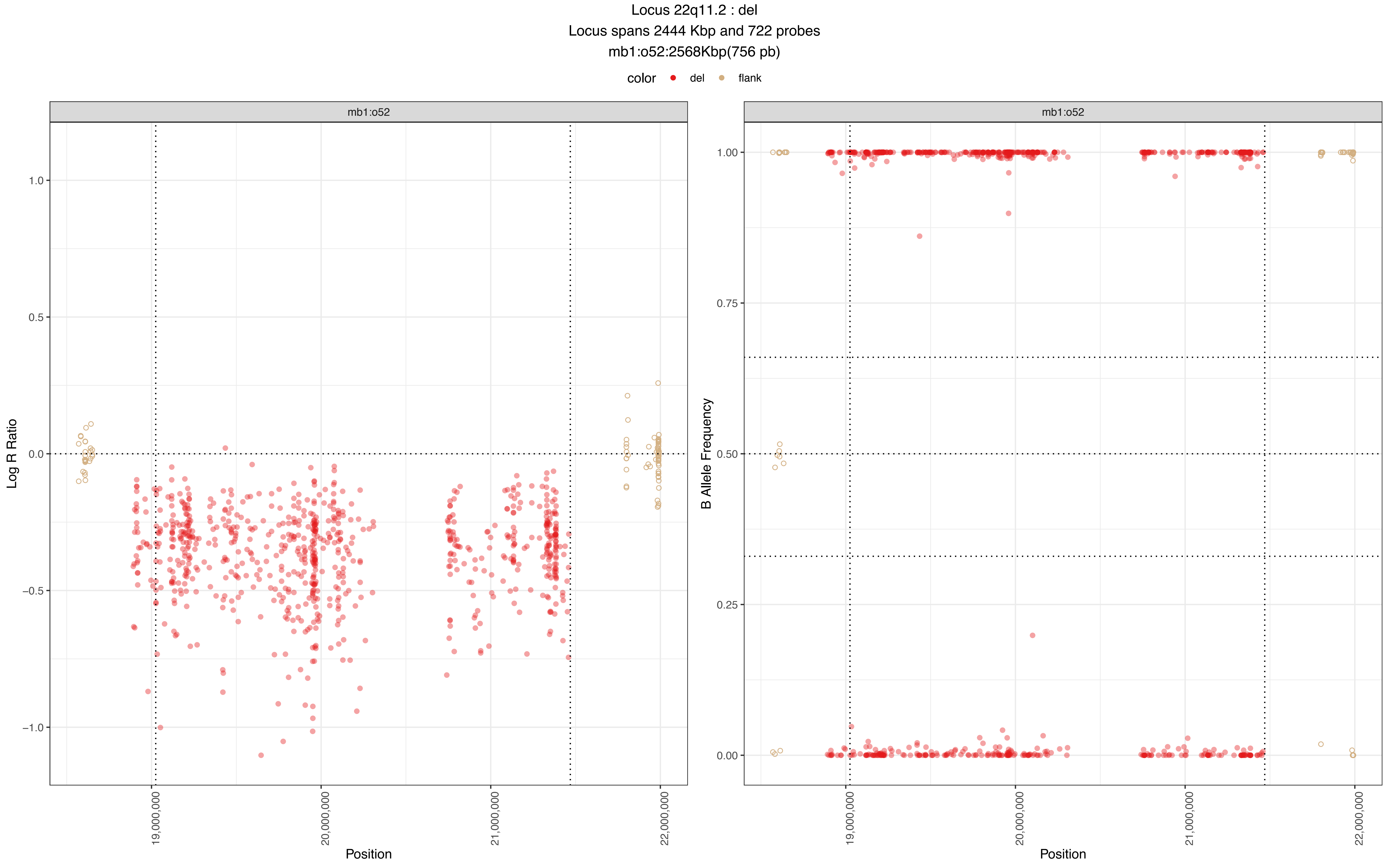

Locus 22q11.2 : del
Locus spans 2444 Kbp and 722 probes
mb1:o52:2568Kbp(756 pb)
color
del
flank
mb1:o52
mb1:o52
Log R Ratio
B Allele Frequency
1.0
0.5
0.0
−0.5
−1.0
1.00
0.75
0.50
0.25
0.00
19,000,000
20,000,000
21,000,000
22,000,000
Position
Position

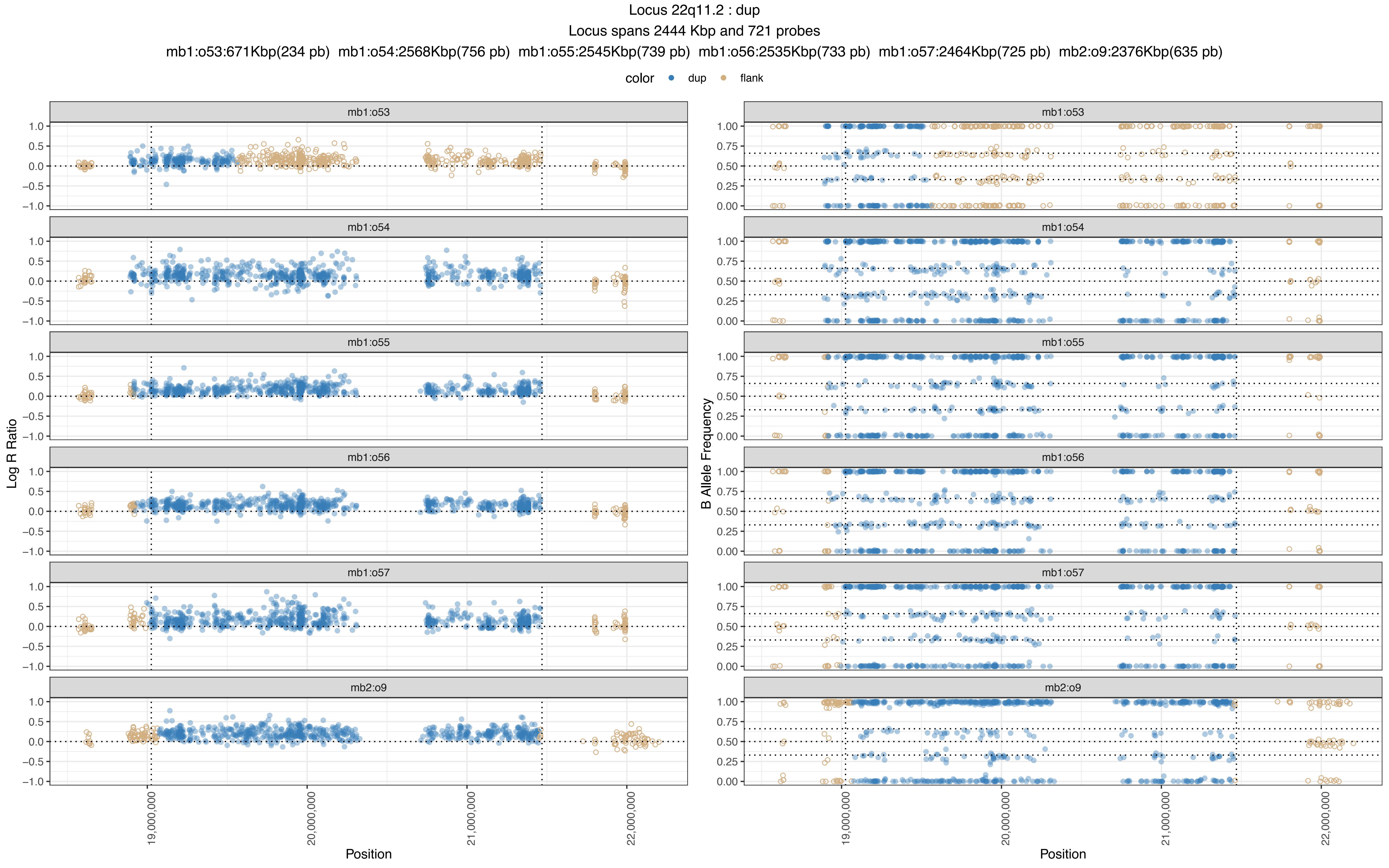

Locus 22q11.2 : dup
Locus spans 2444 Kbp and 721 probes
mb1:o53:671Kbp(234 pb) mb1:o54:2568Kbp(756 pb) mb1:o55:2545Kbp(739 pb) mb1:o56:2535Kbp(733 pb) mb1:o57:2464Kbp(725 pb) mb2:o9:2376Kbp(635 pb)
color dup flank
mb1:o53
mb1:o54
mb1:o55
mb1:o56
mb1:o57
mb2:o9
Log R Ratio
B Allele Frequency
Position
19,000,000
20,000,000
21,000,000
22,000,000
